# Olive Polyphenol Oxidase Gene Family

**DOI:** 10.3390/ijms24043233

**Published:** 2023-02-06

**Authors:** Rosario Sánchez, Laura Arroyo, Pilar Luaces, Carlos Sanz, Ana G. Pérez

**Affiliations:** Department of Biochemistry and Molecular Biology of Plant Products, Instituto de la Grasa—Spanish National Research Council (IG-CSIC), 41013 Seville, Spain

**Keywords:** *Olea europaea* L., polyphenol oxidase, tyrosinase, phenolic metabolism, hydroxytyrosol

## Abstract

The phenolic compounds containing hydroxytyrosol are the minor components of virgin olive oil (VOO) with the greatest impact on its functional properties and health benefits. Olive breeding for improving the phenolic composition of VOO is strongly dependent on the identification of the key genes determining the biosynthesis of these compounds in the olive fruit and also their transformation during the oil extraction process. In this work, olive polyphenol oxidase (PPO) genes have been identified and fully characterized in order to evaluate their specific role in the metabolism of hydroxytyrosol-derived compounds by combining gene expression analysis and metabolomics data. Four PPO genes have been identified, synthesized, cloned and expressed in *Escherichia coli*, and the functional identity of the recombinant proteins has been verified using olive phenolic substrates. Among the characterized genes, two stand out: (i) *OePPO2* with its diphenolase activity, which is very active in the oxidative degradation of phenols during oil extraction and also seems to be highly involved in the natural defense mechanism in response to biotic stress, and (ii) *OePPO3*, which codes for a tyrosinase protein, having diphenolase but also monophenolase activity, which catalyzes the hydroxylation of tyrosol to form hydroxytyrosol.

## 1. Introduction

Virgin olive oil (VOO), which is extracted solely by physical methods from the olive fruit (*Olea europaea* L.), is the main lipid source in the Mediterranean diet. The well-known health benefits associated with VOO are related to its high content of monounsaturated fatty acids—mainly oleic acid, accounting for up to 80% of its total fatty acids—but to a greater extent with the presence of bioactive compounds included in the fraction of the so-called minor components, which have contents much higher than those of other vegetable oils [1]. Among them, phenolic compounds are undoubtedly the minor components with the greatest impact on the functional properties of VOO [2].

The main phenolic components of VOO are the secoiridoid derivatives of hydroxytyrosol (HTy) and tyrosol (Ty), with the hydroxytyrosol-containing phenolic components having the highest biological activities. They are the most effective in preventing chronic diseases due to their superior ability to reduce chronic inflammation and oxidative damage and their potent anti-proliferative activity [3,4]. In this sense, scientific evidence on the health benefits of HTy has led the European Food Safety Authority (EFSA) to approve a health claim that states “olive oil polyphenols contribute to the protection of blood lipids from oxidative stress”. However, the application of this claim is restricted to those oils that contain at least 250 ppm of hydroxytyrosol and its derivatives [5]. This EFSA health claim has promoted the development of different lines of research to obtain oils enriched in HTy derivatives, such as a better understanding of the metabolism of phenolic compounds in the olive, through the identification of key enzymes/genes, the development of new extraction technologies that favor the accumulation of phenols [6] or the search for molecular markers that could help olive breeders in the identification of new olive genotypes with improved phenolic profiles.

VOO secoiridoid derivatives containing Ty and HTy are formed during the industrial process to extract the oil by the hydrolysis of the water-soluble phenolic glycosides present in the olive fruit carried out by β-glucosidase activities. However, during this process, these phenolic compounds also undergo an oxidation process, mainly due to enzymatic activities such as polyphenol oxidase (PPO) [7]. Thus, in the last decade, we have established that the main endogenous factors that positively influence the accumulation of phenolic components in VOO are the content of phenolic glycosides and the level of β-glucosidase activity in the olive fruit [8]. We have recently completed the molecular and biochemical characterization of the β-glucosidase gene family in olives [9]. On the other hand, we have also observed that the PPO-catalyzed oxidative degradation of secoiridoid compounds, both secoiridoid glycosides and their hydrolyzed derivatives, limits the final phenolic content of VOO [10]. Despite the key role played by olive PPO proteins in the degradation of phenols during the oil extraction process and the hypothetical involvement of some PPOs in the conversion of Ty to HTy [11,12], there is little knowledge about this PPO gene family in olives.

The PPO family is made up of two major classes of enzymes, tyrosinases and catechol oxidases. Tyrosinases catalyze the hydroxylation of monophenols, forming ortho-diphenols that are subsequently oxidized to quinones, having both monophenolase and diphenolase activities, while catechol oxidases have only diphenolase activity. There is a significant number of studies focused on finding structural or mechanistic differences between the enzymes classified in one group or another. However, the identification of the amino acids that act as activity controllers in these proteins is very recent [13]. On the other hand, a variety of kinetic and molecular properties have been described in different plant species in which PPOs are presumably associated with defense mechanisms that are triggered when cell disintegration caused by wounding or mechanical damage allows these chloroplast-located proteins to come into contact with phenolic compounds stored in the cell vacuoles [14].

Previous studies to investigate the role of PPO in the oxidation of phenolic compounds in olive have only described diphenolase activity without ever demonstrating the existence of tyrosinase enzymes [10,15]. None of these previous studies have reported the identification or characterization of the olive PPO genes or their encoded proteins, probably due to the lack of a suitable expression system for the production of soluble and active plant PPOs that was first developed by Dirk-Hofmmeister et al. [16]. The aim of this work was to identify and characterize olive PPO genes and to evaluate their specific role in the biosynthesis and degradation of hydroxytyrosol derivatives by combining gene expression analysis and metabolomics data.

## 2. Results and Discussion

### 2.1. Polyphenol Oxidase Gene Family in Olive

Using the genetic tools previously generated from seven olive cultivars that present highly contrasted contents of phenolic compounds [17], fifty proteins annotated as polyphenol oxidases have been identified, which, therefore, could presumably be involved in the biosynthesis or oxidative degradation of olive phenolic compounds. Only seven of them (Olive Genome Data Base accession codes OE6A068152, OE6A114203, OE6A058214, OE6A046766, OE6A063859, OE6A110596 and OE6A053284) showed relevant expression levels (>250 FPKM) and significant differential expression between the olive cultivars and ripening stages used for the generation of the genomic tools (Table S1).

Most of the proteins encoded by these seven transcripts showed a high percentage of pairwise identity (OE6A114203-OE6A058214, OE6A046766-OE6A063859, and OE6A110596-OE6A05328) (Table S2), suggesting a gene duplication event in the PPO gene family. OE6A068152 and OE6A114203 transcripts were identified as the previously described *OePPO1* (GenBank Acc. Number MW038828) and *OePPO2* (GenBank Acc. Number MW038828) genes, respectively [18]. On the other hand, OE6A046766 and OE6A110596 transcripts were selected due to their much higher expression levels compared to their duplicates (Table S2). They were named *OePPO3* (Gen-Bank Acc. Number OL870608) and *OePPO4* (Gen-Bank Acc. Number OM460173), respectively, and together with the *OePPO1* and *OePPO2* genes, they have been molecular and biochemically characterized in this work.

**Figure 1:**
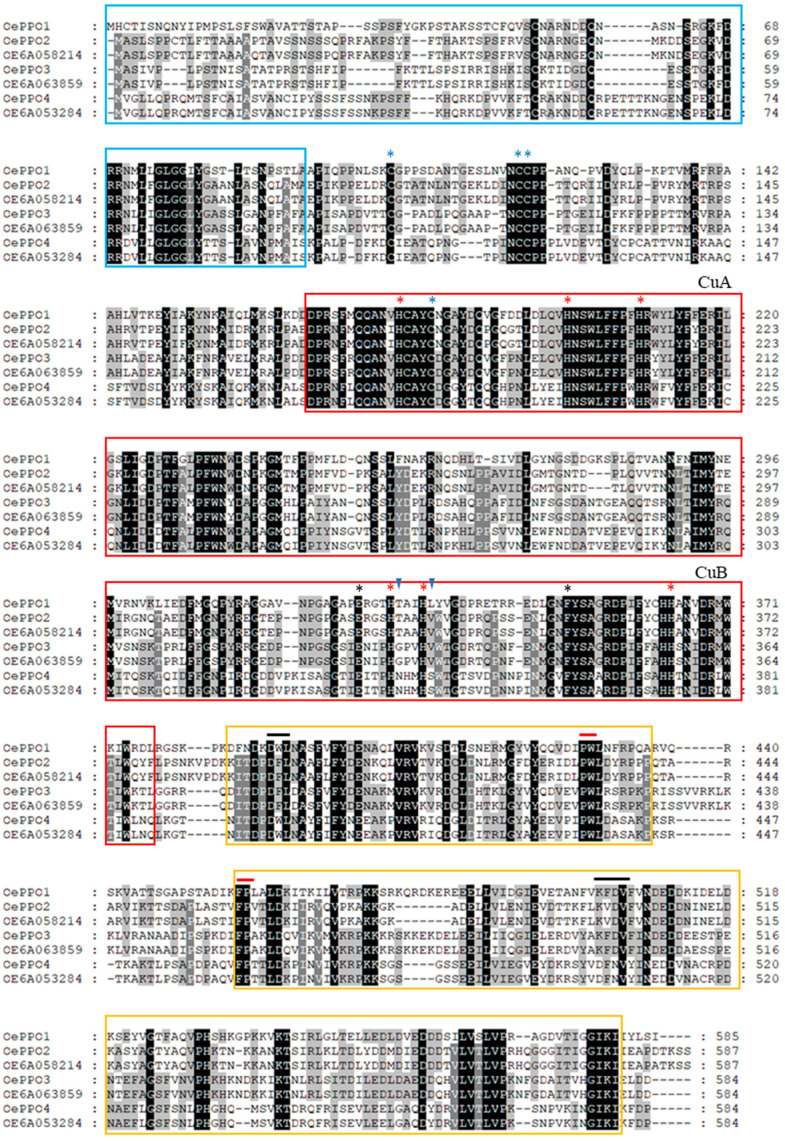
Multiple sequence alignment of the seven olive PPO proteins identified by transcriptomic analysis. Identical and similar residues are shaded in black and grey, respectively. N-terminal chloroplast transit peptide is squared in blue, and the CuA and CuB domains (tyrosinase domain) are squared in red. The DWL and KFDV domains (middle and C-terminal domains, respectively) are squared in orange, showing the corresponding conserved motif (black line) and the amino acids framing the putative proteolytic region of activation (red line). Strictly conserved amino acids are marked with an asterisk: the copper coordinating histidines (red) of the dicopper centre (CuA and CuB), the cysteines (blue) involved in the disulfide bonds and the thioether bridge and the conserved glutamic acid (water-keeper) and phenylalanine (gatekeeper) residues (black). The two amino acids next to the first and second conserved histidine of CuB (activity controllers) are indicated with a blue triangle.

The amino acid sequences of the proteins encoded by the seven olive *PPO* genes were aligned with the help of the ClustalX and GeneDoc programs and subjected to the NCBI Conserved Domain Search and the online subcellular localization tools TargetP2.0 and DeepLoc1.0 (see Section 3 for software details). All the predicted proteins showed the typical structure of plant PPO proenzymes: an N-terminal chloroplast transit peptide, a latent peptide containing the catalytic active domain and a C-terminal domain that shields the active site and keeps the enzyme in a latent state. The analysis of the sequences allowed us to identify a series of domains characteristic of polyphenol oxidases, confirming that the seven genes could have such a function (Figure 1). These domains are: (i) a central domain that consists of the union of two copper ions through two sets of three histidines; (ii) a middle domain of approximately 50 amino acids, also called DWL (the conserved motif); and (iii) a domain of unknown function or domain KFDV (the conserved motif) that is usually located in the C-terminal. Several residues in the vicinity of the active site of PPO enzymes have been related to the different enzyme activities of monophenolase and diphenolase. Thus, a gatekeeper residue, usually a conserved phenylalanine in plant PPOs, theoretically restricts access to the active site but also stabilizes the correct orientation of some substrates. On the other hand, a conserved residue of glutamic acid (waterkeeper) has been described that seems to stabilize the water molecule putatively involved in the deprotonation of the monophenol substrates [19,20]. More recently, two non-conserved residues have also been identified as activity controllers: the first amino acid after the first CuB coordinating histidine (H_B1_ + 1) and the amino acid immediately following the second CuB coordinating histidine (H_B2_ + 1), which could promote or restrict monophenolase activity [13].

### 2.2. Enzymatic Activities of Encoded Olive PPO Proteins

**Figure 2:**
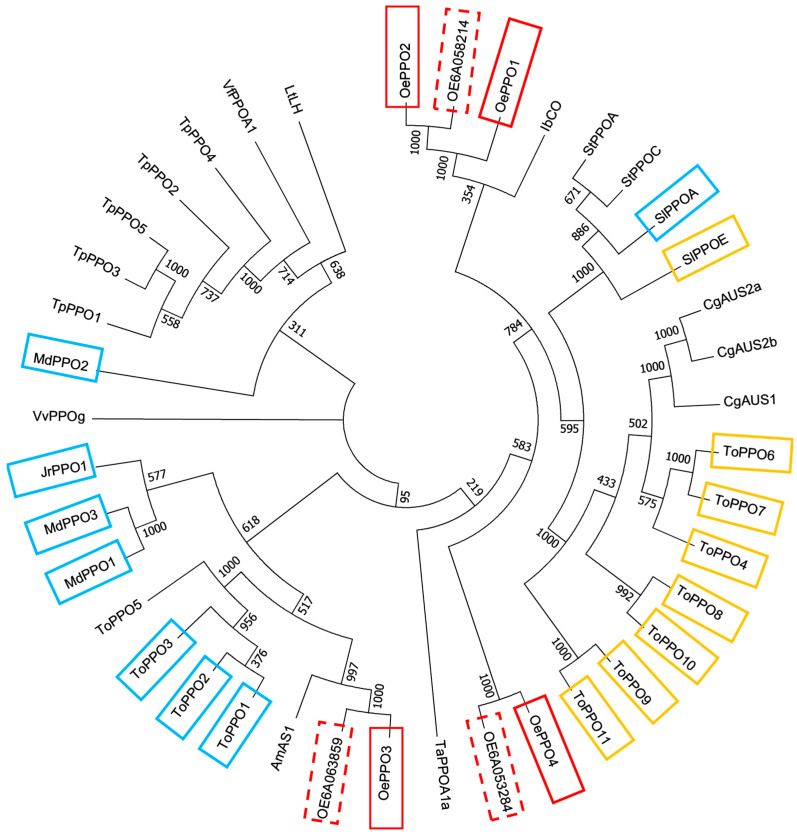
Phylogenetic tree illustrating relatedness of the seven olive *PPO* genes identified by transcriptomic analysis to other plant *PPO* genes. Phylogenetic tree was performed using the neighbor-joining method from MEGA7. Accession numbers are given in Table S2. Red-squared: olive PPOs; solid red line for selected proteins for further studies in this work. Yellow-squared: PPO proteins with diphenolase activity only, as described in the literature. Blue-squared: PPO proteins with both mono- and diphenolase activities as described in the literature.

A phylogenetic analysis of the seven olive PPOs using the sequences of the main PPO characterized so far in plants (Table S3) has been performed. The results obtained showed that olive PPOs could be classified into three different groups (Figure 2). One cluster grouped OePPO1, OePPO2 and its duplicated protein OE6A058214 closer to the *T. officinale* and *C. grandiflora* PPO proteins that only exhibit diphenolase activity [21,22]. A second cluster grouped OePPO3 and its duplicated protein OE6A063859 closer to different plant PPOs exhibiting both mono- and diphenolase activities as those from *M. domestica* [13], *J. regia* [20], and *T. officinale* [21], and finally, the third grouped OePPO4 and its duplicated protein OE6A053284 in a different branch from the other olive PPOs, which are related to other dandelion PPOs that solely exhibit diphenolase activity [21].

The analysis of the activity controller residues flanking the first and second CuB-coordinating histidine residues (H_B1_ and H_B2_) (Figure 1) showed different patterns for the PPO proteins that seem to be correlated with the three different phylogenetic clusters (Figure 2). Thus, OePPO1 and OePPO2 sequences showed threonine and leucine/valine residues in H_B1_ + 1 and H_B2_ + 1, respectively, while OePPO3 had glycine and valine in the same positions that are associated with tyrosinase (both mono- and diphenolase) activity, respectively, in PPO proteins from *T. officinale* [21]. OePPO4 displayed in these positions an asparagine and a serine, being only the first controller, the asparagine, a residue previously associated with tyrosinase proteins from *Malus domestica* (MdPPO2) [13] and *Juglans regia* (JrPPO1) [20].

**Figure 3:**
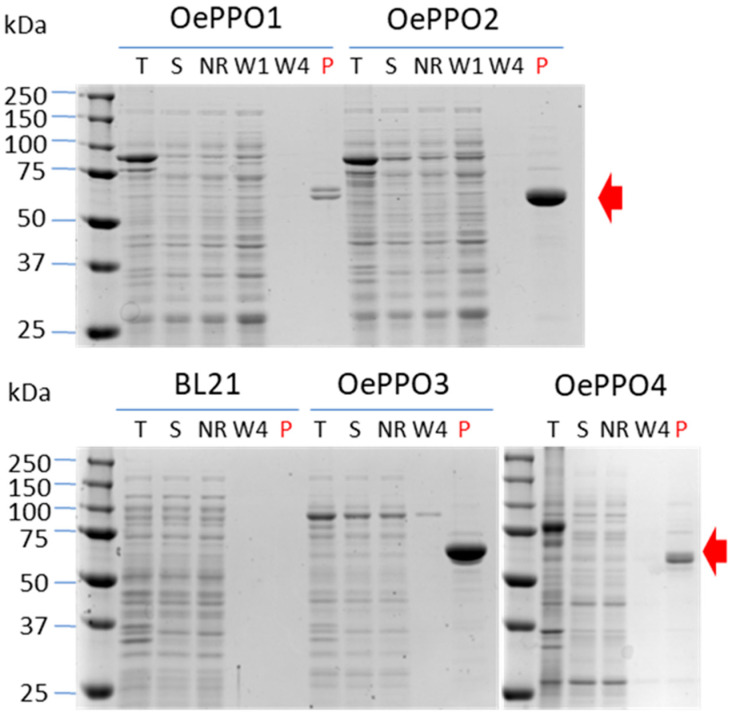
Heterologous expression and purification of the OePPO proteins. SDS-PAGE followed by a Coomassie blue staining showing the recombinant OePPO purification yield by sepharose-GSH beads affinity chromatography. The results of the inductions for each GST-OePPO recombinant protein (~80 kDa) are shown in lanes T (*E. coli* total fraction) and S (*E. coli* soluble fraction). The red arrow shows the corresponding OePPO purified (P) proteins (~55 kDa) from the soluble fraction after removing the GST fusion protein. BL21 refers to control protein extract from untransformed *E. coli* cells. NR: non-retained fraction; W: wash fraction (1–4 column volumes).

To determine the possible role played by the four olive PPO genes in the biosynthesis and degradation of HTy derivatives, it was first necessary to corroborate the functional identity deduced from the phylogenetic analysis of the four *OePPO* genes by characterizing the type of enzymatic activity, monophenolase and/or diphenolase activity, of their encoded proteins. For that purpose, the four *OePPO* coding sequences were synthesized, cloned, expressed as recombinant proteins in *E. coli,* and purified as described in Section 3. The four genes were shown to be around 1750 bp long, encoding proteins of around 585 amino acids. Both the estimated MW (near 66 kDa) and isoelectric points (6.0–6.5) of the four proteins were also very similar (Table S4). The four OePPOs proteins were efficiently purified as GST-independent polypeptides, with an approximate molecular mass of 54 kDa, consistent with the expected values for peptides expressed without a signal peptide (Figure 3).

Purified OePPO1 and OePPO4 proteins exhibited a double band in the SDS-PAGE. This electrophoretic profile has been described for other plant PPOs and seems to be associated with the incorrect folding of recombinant PPOs, with the lower band being the correctly folded and active enzyme [16]. On the contrary, OePPO2 and OePPO3 were always purified as single bands under the same conditions. Detailed analysis of diluted OePPO2 enzyme showed that it corresponded to the lower band size compared to purified OePPO1 (Figure S1).

The enzyme activity of the purified OePPOs was initially assayed by means of a spectrophotometric method using two different substrates: 4-tert-butylcatechol (TBC) for diphenolase activity and tyramine for monophenolase activity. OePPO1 and OePPO2 were only active towards TBC, OePPO3 was active towards both TBC and tyramine, and no activity was detected when OePPO4 was assayed with either substrate (Table 1). These data demonstrate that the substrate specificities assigned based on the phylogenetic analysis were correct since activity assays confirmed that OePPO1 and OePPO2 are diphenolase proteins while OePPO3 belongs to the tyrosinase family, having both monophenolase and diphenolase activities. It seems reasonable that the proteins encoded by the previously mentioned duplicated genes exhibit PPO activity equivalent to those described for OePPO1, OePPO2 and OePPO3. The pH and temperature optima of these three active recombinants proteins were quite similar. The optimum pH was around 6.8–7.0, and the optimum temperature was around 25 °C (Table 1). The enzymes appear to be more stable at basic pHs than at acidic pHs, with 50% decreases in activity being observed below pH 6. Similar pH dependences have been reported for other plant PPOs, such as those from grape [12], apricot [23] or tomato [19]. Active OePPO proteins also proved to be quite thermolabile since their activity was reduced by half when reactions were carried out at 50 °C. In general, all OePPOs were very unstable proteins that lost around 75% of their activity after 72 h of storage at 4 °C, even when glycerol was used as a stabilizer. Kinetic characterization studies were carried out with the diphenolase protein OePPO2 (OePPO1 was discarded due to its lower purification yield) using TBC as a substrate and with the tyrosinase protein OePPO3 using tyramine and TBC as substrates (Table 1).

The *K*_M_ values calculated for the different substrates seem to indicate that OePPO3 was more active as diphenolase than OePPO2, having both a lower *K*_M_ for TBC and also a higher *V*_max_. On the other hand, although the *K*_M_ calculated for OePPO3 with tyramine and TBC substrate (0.7 mM and 0.8 mM, respectively) indicates a slightly higher affinity for the monophenolic substrate, the *V*_max_/*K*_M_ coefficient indicates a clearly higher catalytic efficiency of OePPO3 in the diphenolase reaction. A similar behavior was found for Red Globe grape PPO, also involved in Ty and HTy metabolism, which was reported to have lower specificity for HTy than for Ty but a significantly higher *V*_max_ and catalytic efficiency [12].

**Figure 4:**
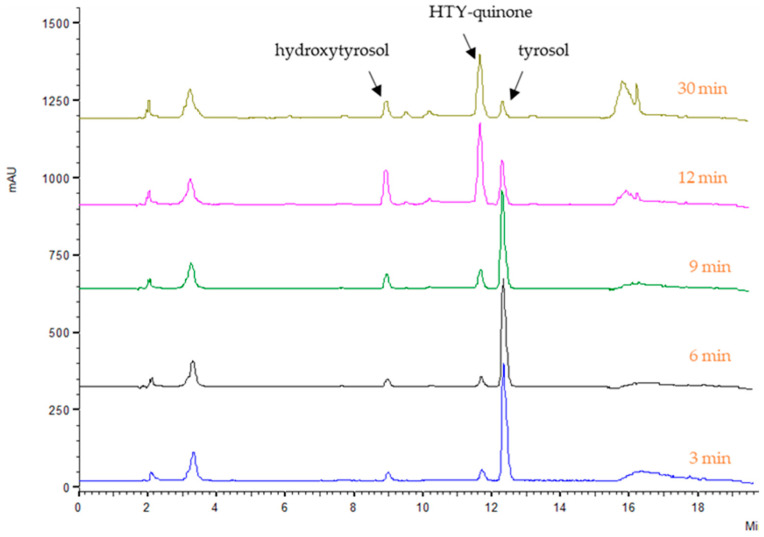
Conversion of tyrosol (Ty) to hydroxytyrosol (HTy) and the hydroxytyrosol quinone (HTy-quinone) by the recombinant OePPO3 protein.

The substrate specificity deduced by the spectrophotometric assays was also verified using olive natural mono- and diphenolic substrates (Ty and HTy). Analysis of the oxidative degradation of Ty and HTy and the formation of the corresponding quinone was carried out by HPLC according to the methodology described in the experimental section. Thus, OePPO1 and OePPO2 were shown to be active with HTy, catalyzing the oxidative degradation of this compound and forming the corresponding quinone, but did not show activity towards a monophenol such as Ty (Figure S2). On the contrary, OePPO3 protein was active against both the mono- and diphenolic substrates, catalyzing the conversion of Ty to HTy and also the oxidation of HTy, acting as a typical tyrosinase. Figure 4 shows the kinetics of the reaction catalyzed by OePPO3 with Ty as substrate. No activity was detected with OePPO4 under the same conditions with either Ty or HTy. The explanation of why some PPO proteins have monophenolase and diphenolase activity while others only have the latter has been studied over the years, with no clear consensus as to which are the key determinants.

### 2.3. In Silico OePPOs Modelling and Substrates Docking 

**Figure 5:**
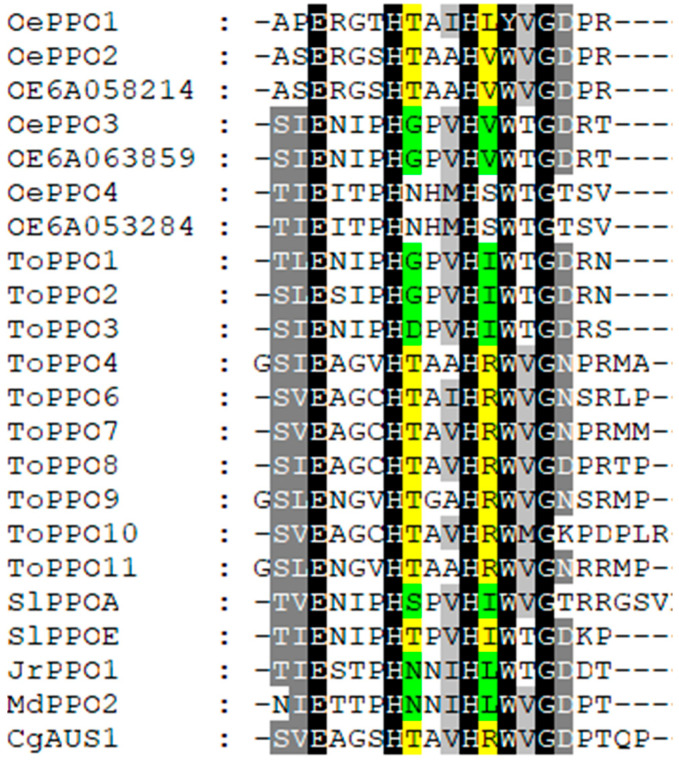
Multiple amino acids alignment of plant PPO regions containing the two activity controller residues, H_B1_ + 1 and H_B2_ + 1. Identical and similar residues are shaded in black and grey, respectively. Residues from PPOs with diphenolase activity only have a yellow background, and those from PPOs with di- and monophenolase activities are in green. GenBank Acc. Numbers for plant PPOs are described in Table S3.

The biochemical characterization of the active OePPOs previously described confirms the existence of two subfamilies of PPO in olive: tyrosinase (OePPO3) and diphenolase (OePPO1 and OePPO2) proteins. This classification correlated with the differences found in the sequences, especially in relation to residues flanking the first and second CuB-coordinating histidine residues (H_B1_ and H_B2_). Figure 5 shows the alignment of the partial sequences of plant PPOs already characterized as monophenolase or diphenolase proteins containing the activity controller residues (H_B1_ + 1 and H_B2_ + 1). OePPO1 and OePPO2 showed a threonine in H_B1_ + 1 position, which has also been found in *C. grandiflora* aurone synthase (CgAUS1) [22], the tomato SlPPOE [19] and in all the ToPPO proteins with diphenolase activity [21]. The oePPO3 sequence contains a glycine residue that has also been found in two tyrosinases from dandelion, ToPPO1 and ToPPO2 [21]. On the other hand, despite its lack of activity, the OePPO4 sequence showed an asparagine residue that has been reported in previously characterized proteins from apple (MdPPO2) [13] and walnut (JrPPO) [20] having tyrosinase activity.

**Figure 6:**
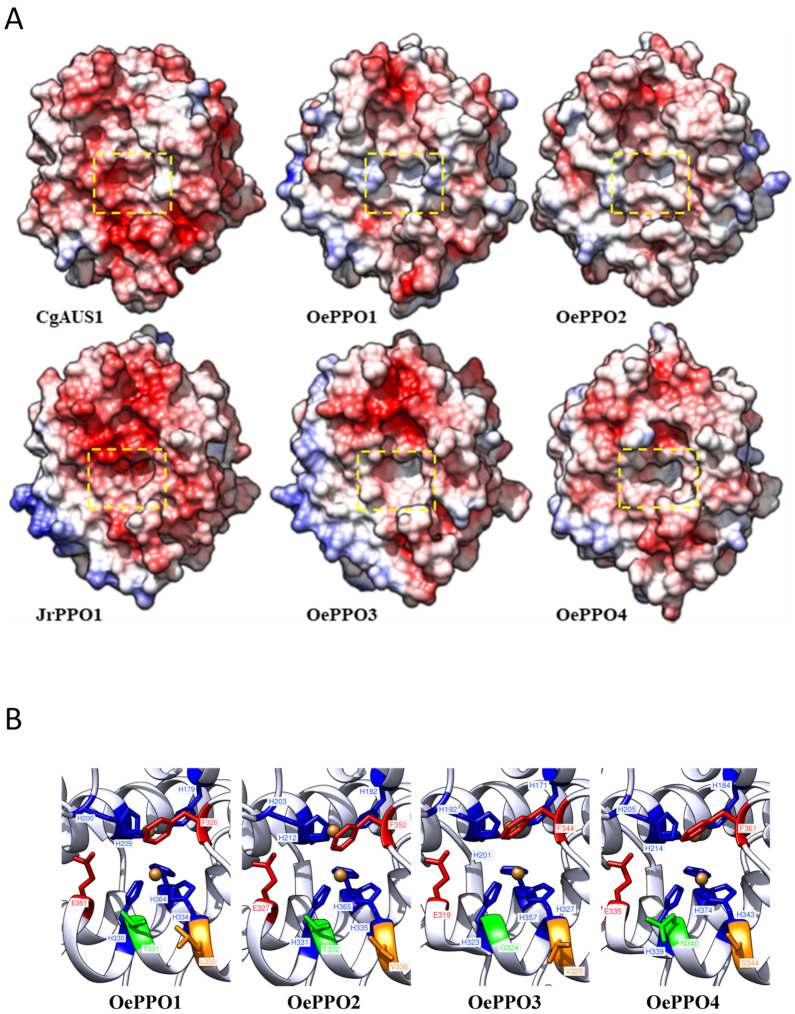
Molecular 3D structure of olive PPOs. (**A**) Coulombic surface coloring for olive PPOs’ molecular 3D structures and their references for modelling. Positively charged amino acid residuals are shaded blue, and negatively charged ones are in red. Activity pocket of the olive PPOs and the proteins used for their molecular modelling are in yellow squares. (**B**) Active centers of the olive PPOs. Highlighted amino acids in all the enzymes: the six conserved histidines coordinating CuA and CuB (gold spheres) are highlighted as blue sticks; the gatekeeper (phenylalanine) and waterkeeper (glutamic acid) residues are highlighted as red sticks; the first activity controller (H_B1_ + 1) is shown in green, and the second activity controller (H_B2_ + 1) is displayed in orange. OePPO1 and OePPO2 are shown as homology models to the CgAUS1 (PDB ID:4z11), and OePPO3 and OePPO4 are shown as homology models to JrPPO1 (PDB ID:5ce9).

We further studied the catalytic pocket to obtain clues for the substrate selectivity of OePPO proteins. Three-dimensional structures for the olive PPOs were constructed using the Modeller v10.2 tool and the UCSF Chimera v1.16 software (see Section 3 for software details) based on the crystal structures of *C. grandiflora* aurone synthase (CgAUS1; PDB ID: 4z11) as a template for OePPO1 (48% of identity) and OePPO2 (47% of identity) and *J. regia* tyrosinase (JrPPO; PDB ID: 5ce9) for the OePPO3 (68% of identity) and OePPO4 (52% of identity). Although all olive PPOs showed an identity percentage above 50% to the JrPPO1 for the core sequence (starting after the signal peptide and ending in the proteolytic site), we chose to model OePPO1 and OePPO2 using the CgAUS1 due to the similarity of their diphenolase activity. The whole backbone conformation of the resulting models was very similar to that of the template resulting in the catalytic pocket with a similar shape to their corresponding models, OePPO1 and OePPO2 to CgAUS1 and OePPO3 to JrPPO1, except for OePPO4, which showed a pocket more closed than its model (Figure 6A). OePPO1 and OePPO2 displayed a pocket with a defined opening in contrast with OePPO3, which displayed a more open pocket, and OePPO4, which showed a tighter one. A closer look at the activity pockets showed the positions of all the main residues involved in the catalytic site (Figure 6B), suggesting that the locations of the activity controller residues are responsible for the shape of the bottom of the catalytic gap, as supported by the 3D structure for each PPO in which these residues are labelled. The absence of activity detected in recombinant OePPO4 could be related to the smaller size of the catalytic pocket found for this protein, whose shape could also be affected by a serine residue at the H_B2_ + 1 position, which has not been previously described in another plant PPO.

The activity controller residues do not support substrate selectivity on their own, as described by Prexler et al. [21], who failed to transform the catechol oxidase ToPPO6 into a tyrosinase by simple mutations of these residues. Thus, according to previous studies, activity controller residues are not the unique feature that entails the activity with mono- or diphenol substrates but the set of amino acids that make up the catalytic cavity [13,16,19]. The presence of negatively and positively charged amino acids in the positions of activity controllers can reduce or inhibit the monophenolase activity by affecting the basic character of the water molecule that carries out the deprotonation of the monophenol substrate [13]. Bulky residues in the hydrophobic catalytic cavity could also play a role in reducing the size of the opening to the hydrophobic active cavity [16] or the substrate stability [19]. Active olive PPOs did not show any charged amino acid in these positions, but residues with different hydrophobicities and volumes at the H_B1_ + 1 position, such as a small and non-polar glycine in the tyrosinase OePPO3 versus a bulky and polar threonine residue presented in both catechol oxidases OePPO1 and OePPO2. Furthermore, OePPO1 showed a major presence of negatively charged amino acids in the vicinity of the catalytic pocket (Figure 6A) that could explain the differences in specific activity between both olive catechol oxidases (Table 1) that share a threonine in the H_B1_ + 1 position and had similar residues, valine and leucine, respectively, in H_B2_ + 1.

**Figure 7:**
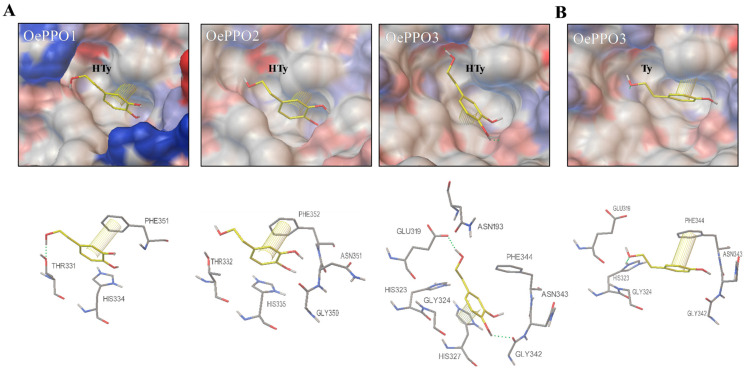
Docking analysis of olive PPOs. (**A**) Potential docking of the diphenolic substrate hydroxytyrosol (HTy) into the active site of OePPO1, OePPO2 and OePPO3 proteins. (**B**) Potential docking of the monophenolic substrate tyrosol (Ty) into the active site of the OePPO3 enzyme. Surface views of the active pockets are coloured by polarity, with red for negative and blue for positive charged amino acid residues. Amino acids involved in the interaction with the substrate are shown under each docking pose. Hydrogen bonds are shown as green dashed lines, and π-π interactions between aromatic rings are shown as yellow lines.

A docking analysis of the Hty and Ty molecules into the active site of the corresponding olive enzymes to which they act as substrates is shown in Figure 7. The interacting amino acids and the surrounding polarity may all contribute to allowing the chemical reaction to take place. This way, hydrogen bonds and hydrophobic interactions between the π-electron system of the phenolic rings may lead to the correct positioning and stabilization of the substrate into the active site [21]. As mentioned above, OePPO1 displayed fewer hydrophobic residues closer to the active pocket, which could be responsible for a lower interaction of the substrate with the active site residues leading to a lower stabilization and enzymatic activity (Figure 7A, first panel). Thus, the highest number of amino acid interactions to HTy shown by the OePPO3 active site residues (Figure 7A) could explain its higher diphenolase activity compared to OePPO2 (Table 1).

Figure 7B shows a pose for the Ty substrate in the olive tyrosinase OePPO3 catalytic pocket, where the substrate interacts with the glutamic residue E-319 (water-keeper), the phenylalanine F-344 (gatekeeper) and the substrate specificity controller G324 (H_B1_ + 1) among others. In all docking poses shown in Figure 7, the phenylalanine acting as the gatekeeper and the corresponding H_B1_ + 1 residue, T331 (OePPO1), T332 (OePPO2) and G324 (OePPO3), are involved as expected to enable the access to the catalytic site and the substrate selector functions, respectively.

### 2.4. Gene Expression Analysis of Olive PPOs

#### 2.4.1. OePPOs Expression Is Cultivar Dependent

**Figure 8:**
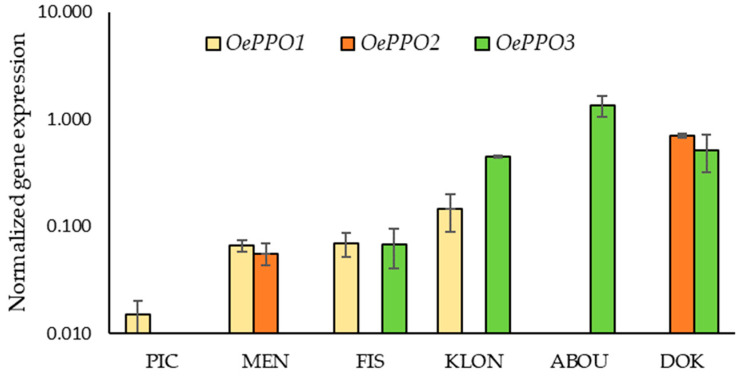
Relative expression levels of olive *OePPO1*, *OePPO2* and *OePPO3* genes in the mesocarp tissue of ‘Picual’, ‘Menya’, ‘Fishomi’, ‘Klon’, ‘Abou Kanani’, and ‘Dokkar’ fruits harvested at the usual ripening stage used for olive oil extraction (stage III).

The relative expression levels *of OePPO1, OePPO2* and *OePPO3* genes were studied in the fruit mesocarp of six olive cultivars with marked differences in terms of phenolic contents. For this purpose, fruits harvested at ripening stage III (fully colored fruits) were used, a stage usually used for VOO extraction (Figure 8). Expression levels measured by RT-qPCR, as described in the Section 3, were quite low in most olive cultivars, confirming the absolute expression levels previously obtained in the transcriptomic analysis (Table S1). Among the cultivars tested, ‘Abou Kanani’ and ‘Dokkar’ had the highest values, and ‘Picual’ had the lowest values.

Considering that, as mentioned above, the main endogenous factors that positively affect the accumulation of phenolic components in VOO are the content of phenolic glycosides and the level of β-glucosidase activity in the olive fruit, the role played by olive PPOs in determining the phenolic content of VOO was studied. For this, the expression levels of the olive PPO genes were analyzed together with those of the main β-glucosidase gene (*OeBGLU1A*) involved in the conformation of the phenolic profile of the oil [9] and the phenolic content of the fruits and the corresponding oils (Figure S3).

It has been found that, despite the great influence of the phenolic content of the fruits and the β-glucosidase activity on the phenolic profile of VOO, in some olive cultivars, the expression levels of *OePPO* genes are key to explaining the phenolic content of the oils. Thus, it was expected for cultivar ‘Picual’ to obtain oils with low phenol content according to the medium phenol content of the fruits and the low expression levels of *OeBGLU1A*, the lowest among the cultivars studied. However, the oil obtained from ‘Picual’ fruits had the highest phenolic content among all the oils of the cultivars analyzed (Figure S3). The high phenolic content of ‘Picual’ oils could only be explained by the extremely low expression levels of *OePPO* genes (Figure 8), which means that the oxidation of the phenolic compounds catalyzed by PPO during the oil extraction process is minimal. In contrast, the fruits of the cultivar ‘Dokkar’ contain extraordinarily high contents of phenolic glycosides and have medium expression levels of the *OeBGLU1A* gene, which theoretically should lead to oils with very high phenolic content. However, ‘Dokkar’ oils have a medium/low phenolic content that can only be explained by the high expression levels of *OePPO* genes, which causes a large amount of PPO activity to occur during the oil extraction process, which limits the phenolic content of the oils. Therefore, although the high expression of β-glucosidase genes can be a good marker in breeding programs to select olive genotypes that can produce oils with high phenolic content [9], the expression levels of *OePPO* genes could also have a certain predictive value given that high levels of PPO would always be associated with a high oxidative degradation of phenols and therefore with a reduction in the final phenolic content of the oil.

#### 2.4.2. Olive PPO Genes Are Involved in Plant Defense Mechanisms

As previously mentioned in the introduction section, plant PPOs have been related to natural defense mechanisms [14]. In order to evaluate the role of olive *PPO* genes in the natural defense mechanisms of olives, we have analyzed the expression levels of *OePPO* genes in fruits both infected by *Bactrocera oleae* (olive fruit fly), the most important olive pest, and treated with methyl jasmonate, a volatile plant hormone that induces and mediates plant defensive responses against both biotic and abiotic stressors [24]. In this sense, we have recently seen that the infestation by the olive fly causes a modification of the metabolic profile of the oils, giving rise to oils with a higher content of volatiles but with a significantly lower content of phenolic compounds [18]. 

**Figure 9:**
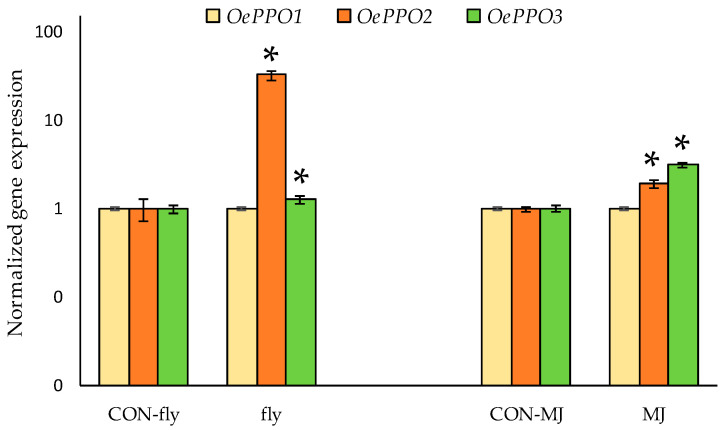
Relative expression of olive PPO genes by RT-qPCR in cultivar ‘Picual’ fruits under different stress conditions (see Section 3). CON-fly: control sample in fly experiment; fly: fly-attacked fruit; CON-MJ: control sample in methyl jasmonate-treated fruits; MJ: methyl jasmonate-treated fruits. Asterisks (*) mean gene expression values are statistically different (*p* < 0.05) from their corresponding control.

Figure 9 shows the expression levels of *OePPO1*, *OePPO2* and *OePPO3* in fruits attacked by the olive fruit fly. 

Gene expression analysis confirmed the involvement of OePPO2 in the plant response as described previously by Notario et al. [18] and suggested a minor role of OePPO3 in defense. Thus, the very high expression of OePPO2 and also the significant increase in OePPO3 would result in an increased oxidative activity during the oil extraction process that would reduce the phenolic content of the corresponding oils [18]. Similarly, the expression levels of these three genes were analyzed in fruits of cultivar ‘Picual’ treated with methyl jasmonate (see Section 3 for treatment details). The results show less alteration of the expression levels, being two and three times higher for OePPO2 and OePPO3, respectively, than in the control samples. The results obtained in both experiments suggest the existence of an oxidative defense system mediated by PPO in olives.

## 3. Materials and Methods

### 3.1. Plant Material

Six olive cultivars selected based on their contrasting levels of phenolic compounds in both the fruit and the oils were studied: ‘Picual’, ‘Menya’, ‘Fishomi’, ’Klon’, ‘Abou Kanani’ and ‘Dokkar’. Trees, two per accession, were grown in the same edaphoclimatic conditions at the experimental orchards of Instituto de la Grasa (Seville, Spain). Olive fruits were harvested at stage III of ripening (fully colored fruits).

‘Picual’ olive trees were used for the *Bactrocera oleae* infestation experiments and the treatments with methyl jasmonate (MJ). For the former, around 10 kg of ripening stage III fruits were collected from five different ‘Picual’ trees, immediately transported to the laboratory and divided into two groups: one with healthy fruits with no symptoms of fly attacks and another group of fruits with obvious symptoms of olive fly attacks (larvae and/or pupae and/or exit holes). The full experimental methodology used for the experiments with *Bactrocera oleae* has been exhaustively described in a previous paper [18]. For the MJ experiments, two foliar treatments with MJ were carried out on ‘Picual’ trees before the start of fruit ripening (September 1st) and one month later when the fruits were in the turning stage (half-colored fruit). The treatment consisted of the spray application of a solution of 10 mM MJ and 0.1% Tween 20 on the tree, according to a previously described methodology [25]. Control trees were treated with a solution containing only 0.1% Tween 20. MJ-treated and untreated fruits were harvested at stage III of ripening.

### 3.2. Identification of Putative Olive Polyphenol Oxidase Genes

Putative PPO genes that might be involved in the biosynthesis or oxidative degradation of olive phenolic compounds were selected from genes annotated as PPO in a transcriptomic study performed from seven olive cultivars contrasting in their phenolic contents [17] that was annotated against the olive genome database (OE6.OLIVEFAT, https://denovo.cnag.cat/olive_data, accessed on 26 August 2021). Gene ontology (GO) terms and enrichment analysis were conducted using the Blast2GO software v5.2 with default parameters.

### 3.3. Sequence Alignment and Phylogenetic Analysis

The multiple sequence alignments of plant PPO amino acid sequences were calculated using the ClustalX v2.1 program [26] and displayed with GeneDoc v2.7 (https://genedoc.software.informer.com, accessed on 17 January 2022). The subcellular localizations of the proteins were predicted using the DeepLoc1.0 online tool (http://www.cbs.dtu.dk/services/DeepLoc/, accessed on 16 December 2021), and the presence of some signal peptide at the N-terminus was assessed with TargetP2.0 (http://www.cbs.dtu.dk/services/TargetP/, accessed on 16 December 2021). The conserved domains in the deduced amino acid sequences were analyzed using the NCBI Conserved Domain Database (CDD) [27]. The expected isoelectric point (pI) and molecular mass of the studied proteins were calculated with ExPASy Compute pI/Mw (https://web.expasy.org/compute_pi/, accessed on 17 December 2021).

Phylogenetic tree analysis was performed using the neighbor-joining method from MEGA7 [28]. Accession numbers of the different polyphenol oxidases included in the analysis are listed as supplementary data (Table S3).

### 3.4. OePPO Genes Cloning, Heterologous Protein Expression and Purification

The coding sequences of the selected candidate genes, *OePPO1* (OE6A068152), *OePPO2* (OE6A114203), *OePPO3* (OE6A046766), and *OePPO4* (OE6A110596), were synthesized lacking the chloroplast transit peptide (amino acids 1–92, 1–93, 1–84 and 1–98, respectively) with the *Escherichia coli* codon optimization (GenScript, Piscataway, NJ, USA) and cloned into a pGEX6P.1 vector as *EcoR*I-*Xho*I (*OePPO1-3*) or *BamH*I-*Sal*I (*OePPO4*) fragments. Four constructs were obtained to be expressed as *glutathione-S-transferase* (*GST*) fusion proteins: *GST-OePPO1* to *GST-OePPO4*. Protein expression and purification were carried out according to Kampatsikas et al. [13] with minor modifications for each *OePPO* construct. BL21(DE3) lacIq *E. coli* cells containing the corresponding *OePPO* construct were grown at 37 °C in Luria–Bertani media (LB) with NaCl 0.5 M to OD_600_ of 0.6, then supplemented with 0.5 mM isopropyl-β-D-thiogalactoside (IPTG) and 0.5 mM CuSO_4_ and grown for 20 h at 19 °C. Cells were harvested by centrifugation, suspended in lysis buffer (50 mM Tris-HCl, pH 7.5, 0.2 M NaCl, 1 mM EDTA, 10% glycerol, 1 mM benzamidine, and 1 mM PMSF), and lysed by sonication. The resulting crude protein lysate was clarified by centrifugation prior to chromatographic purification with sepharose-GSH beads (GE Healthcare, Chicago, IL, USA) following the manufacturer’s instructions. The soluble fraction obtained was added to the beads for GST-OePPO purification and incubated for 2 h at 4 °C in a 360° rotator. Then, recombinant protein-bound beads were incubated in a preScission Protease proteolytic digestion buffer (GE Healthcare) (50 mM Tris HCl pH 7.0, 150 mM NaCl, 1 mM EDTA) with 160 U/mL of protease. The enzymatic digestion was carried out overnight at 4 °C on a 360° rotator to eliminate the GST fusion protein from the OePPO proteins. The purified OePPO proteins were collected as eluates after centrifugation. The purified protein buffer was exchanged to 100 mM Tris-maleate, pH 6.8, and 10% glycerol buffer on a PD-10 column (Sephadex G-25, GE Healthcare, Chicago, IL, USA) and further concentrated using a Vivaspin centrifugal concentrator (MWCO 30 kDa, Merck, Darmstadt, Germany). The purity of each of the recombinant proteins was evaluated by SDS-PAGE, and the protein concentration was determined by the Bradford assay [29]. The control protein extract consisted of untransformed *E. coli* BL21 cells grown and subjected to the same purification steps.

### 3.5. Polyphenol Oxidase Activity Assays

#### 3.5.1. HPLC Method

Activity assays for the functional characterization of purified recombinant PPO proteins with their natural olive substrates (Ty and HTy) were monitored by HPLC. PPO activity assays were performed in 150 µL of reaction buffer (50 mM sodium phosphate buffer, pH 6.8, 1 mM SDS) containing 10–20 µg of recombinant protein and 2.5 mM of the phenolic substrate Ty (Sigma-Aldrich, St. Louis, MO, USA) or HTy (Extrasynthese, Genay, France). The mixture was incubated for 5–30 min at 25 °C, and then the reaction was terminated by the addition of 150 µL methanol. The reaction mixture was centrifuged and filtered (0.45 µm), and the filtrate was analyzed by HPLC using the same equipment and chromatographic conditions described below for the analysis of phenolic compounds. In all cases, controls were carried out using the same reaction mixture but using untransformed *E. coli* BL21 protein extracts.

#### 3.5.2. Spectrophotometric Method

PPO diphenolase activity was determined by continuously monitoring the increase in absorbance at 400 nm related to the oxidation of tert-butylcatechol (TBC) and the formation of the corresponding quinone. The quantification of the oxidative reaction was carried out considering an extinction coefficient of 1200 M^−1^ cm^−1^ [30]. The reaction medium consisted of 1.5 mL of 50 mM sodium phosphate buffer, pH 6.8, 1 mM SDS, 7 mM TBC, and the appropriate amount of recombinant protein (10–20 µg). Blank reactions with thermally denatured enzyme extracts (60 min at 100 °C) were measured in parallel to quantify and subtract any possible TBC non-enzymatic oxidation. Parallel control reactions were also carried out with protein extracts from untransformed *E. coli* BL21. One unit of diphenolase activity was defined as the amount of enzyme forming 1 μmol of TBC-quinone per minute. PPO monophenolase activity was determined in a similar way using tyramine as a substrate. The quantification of the oxidative reaction was carried out by monitoring the increase in absorbance at 480 nm, considering an extinction coefficient of 3300 M^−1^ cm^−1^ [13]. The optimum pH was determined using sodium acetate, phosphate, and borate buffers (50 mM) in the standard assay. The optimum temperature was determined using a temperature interval of 20–60 °C in the standard spectrophotometric assay. Activity assays for the functional characterization of purified recombinant PPO proteins were carried out at pH 6.8 and 25 °C. To obtain the kinetic parameters, recombinant olive PPO enzyme activity was measured over a range of concentrations (0.2–7 mM) with TBC and tyramine.

### 3.6. Olive Oil Extraction

VOO was extracted using an Abencor analyzer (Comercial Abengoa, S.A., Seville, Spain) that simulates the industrial process of VOO production on a laboratory scale. The processing parameters have been described in a previous study [9].

### 3.7. Extraction and Analysis of Phenolic Compounds from Olive Fruit and Oil 

Fruit phenolic compounds were extracted according to a previously developed protocol [31]. Longitudinal pieces of mesocarp tissue were cut from 20 olive fruits and kept at 4 °C for 72 h in dimethyl sulfoxide (6 mL/g fruit) containing syringic acid (24 mg/mL) as an internal standard. The extracts were filtered through a 0.45 μm nylon filter and kept at −20 °C until HPLC analysis.

VOO phenolic compounds were isolated by solid phase extraction (SPE) on a diol-bonded phase cartridge (Supelco, Bellefonte, PA, USA) based on the method by Mateos et al. [32] and using *p*-hydroxyphenyl-acetic and *o*-coumaric acids as internal standards.

Phenolic extracts were analyzed as described by García-Rodríguez et al. [11] in a Beckman Coulter liquid chromatographic system equipped with a diode array detector and a Superspher RP 18 column (4.6 mm i.d. × 250 mm, particle size 4 µm, Dr. Maisch GmbH, Ammerbuch, Germany). Phenolics were monitored at three different wavelengths, 280, 335 and 517 nm, and quantified considering the internal standard and calibration curves for each of them. The identification of phenolic compounds was established based on their UV–Vis spectra and confirmed by HPLC/ESI-qTOF-HRMS using available standards and similar separation conditions.

### 3.8. Modelling 3D Structures and Molecular Docking

The three-dimensional structures of the OePPO proteins were generated with the Modeller v10.2 tool [33] based on the crystal structures of Coreopsis grandiflora aurone synthase (CgAUS1; PDB ID: 4z11) for OePPO1 and OePPO2 (48% and 47% of identity, respectively) and Juglans regia tyrosinase (JrPPO; PDB ID: 5ce9) for OePPO3 and OePPO4 (68% and 52% of identity, respectively). The modeller tool was performed with the UCSF Chimera v1.16 software [34], which was also used to visualized the 3D structures.

Molecular docking was performed using Autodock Vina v1.2.3 [35] to identify the binding poses of the monophenolic Ty and the diphenolic HTy substrates within the active center of the olive PPOs. The substrates for docking were obtained from the PubChem Structure and Search database (https://pubchem.ncbi.nlm.nih.gov/, accessed on 18 February 2022; Ty, ID 10393; HTy, ID 82755) and prepared for docking, minimizing their energies with the Avogadro v1.2.0 software [36]

### 3.9. Total RNA Extraction and Gene Expression Analysis by Real-Time Quantitative PCR (RT-qPCR)

Total RNA extraction from olive mesocarp tissues was performed using the Spectrum Plant Total RNA Kit (Sigma-Aldrich, St. Louis, MO, USA) according to the supplier’s instructions. The corresponding cDNAs were synthesized using the Ready-To-Go T-Primed First Strand Kit (Amersham Bioscience, Roosendaal, The Netherlands). The cDNAs were subjected to RT-qPCR with specific pairs of primers for the three active olive polyphenol oxidases (*OePPO1* to *OePPO3*) and using SYBR Green I (SsoAdvancedTM Universal SYBR Green Supermix, BioRad, Hercules, CA, USA) in a CFX96 Touch System (BioRad) to monitor the resulting fluorescence. The reaction mixture was heated to 95 °C for 30 s before being subjected to 40 PCR cycles consisting of 95 °C for 15 s, 54 °C for 15 s, and 60 °C for 30 s. Efficiency curves were drawn for each pair of primers using serial dilutions of cDNA. In addition, a thermal cycler melting curve analysis was performed, which resulted in single product-specific melting temperatures. The Pfaffl method [37] was applied using the BioRad CFX Maestro 1.0 Software (BioRad) to calculate the relative expression levels between samples. Two olive genes, elongation factor-1-alpha (*OeEF1α*) and glyceraldehyde-3-phosphate dehydrogenase (*OeGAPDH*) (Olive Genome Database annotation number OE6A045598 and OE6A105640, respectively; https://denovo.cnag.cat/olivedata; accessed on 26 August 2021) were selected as reference genes according to previous validation studies [17]. Specific pairs of primers for these reference genes and *OePPO1-3* are described in Table S5. Three biological and two technical replicates were obtained from each sample. Statistical significance was set at a level of *p* < 0.05 (Student’s *t*-test).

## 4. Conclusions

Due to the important role that PPO activity can play in the conformation of both the phenolic glycosides present in the olive fruit, through the hydroxylation of Ty to HTy, as well as in the oxidative degradation of the derivatives of these compounds during the olive oil extraction process, the PPO gene family that encode these proteins has been studied during the ripening process of this fruit. To do this, taking advantage of the genomic tools developed with a selection of olive cultivars that present very contrasting phenolic contents, a series of genes annotated as PPO have been identified that could potentially intervene in the processes described above. The catalytic characteristics of the proteins encoded by these genes corroborate those deduced by phylogenetic and structural analysis, indicating the existence of two subfamilies of PPO genes. On the one hand, tyrosinases (*OePPO3*) could be involved both in the synthesis of HTy by hydroxylation of Ty and in the oxidative degradation of phenols during the olive oil extraction process; on the other hand, the diphenolases could participate together with the tyrosinases in this last process. It has been found that one of the diphenolase genes (*OePPO2*) is induced after infestation with the olive fruit fly, which suggests that this subfamily of genes would be involved in the olive defense mechanism against biological stressors. Therefore, olive PPO genes could be studied as candidates to be used in marker-assisted olive breeding programs to select olive genotypes that can produce oils with improved functional properties.

## Figures and Tables

**Table 1:** Catalytic properties of olive PPO proteins.

	Substrate	Specific Act. (U/mg)	Optimum pH	Optimum Temp. (˚C)	*K*_M_ (mM)	*V*_max_ (U/mg)	*V*_max_/*K*_M_
OePPO1	TBC	11.1	7.0	25			
	tyramine	nd					
OePPO2	TBC	44.2	7.0	25	12.7	41.6	3.3
	tyramine	nd					
OePPO3	TBC	96.8	6.8	25	0.8	129.9	162.4
	tyramine	1.7			0.7	2.7	3.9
OePPO4	TBC	nd					
	tyramine	nd					

nd: non-detected activity.

## Data Availability

Data is contained within the article.

## Supplementary Materials

The following supporting information can be downloaded at: https://www.mdpi.com/article/10.3390/ijms24043233/s1.
