# Supplementary material for: Olive Polyphenol Oxidase Gene Family
Source: Int J Mol Sci. 2023 Feb 6;24(4):3233. doi: 10.3390/ijms24043233 (PMC9962951; doi:10.3390/ijms24043233)
Supplement: Supplementary file 1 [file ijms-24-03233-s001.zip › ijms-2199211-supplementary.pdf]

Supplementary Materials:

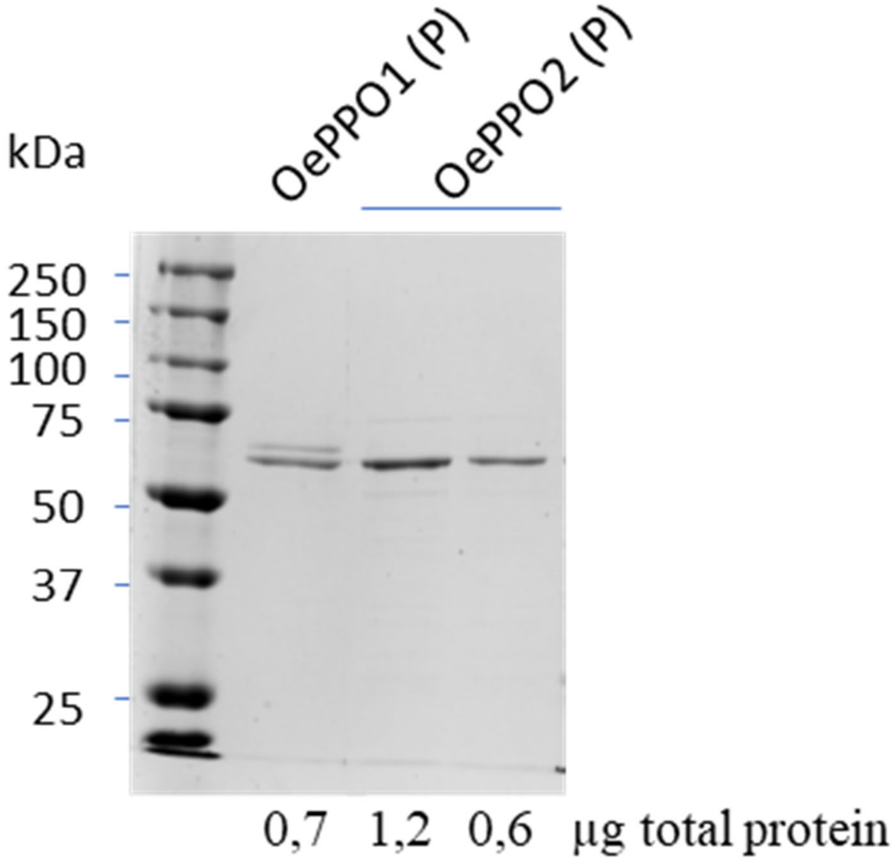

Figure S1. Coomassie stained SDS-PAGE gel showing a double band in purified OePPO1.

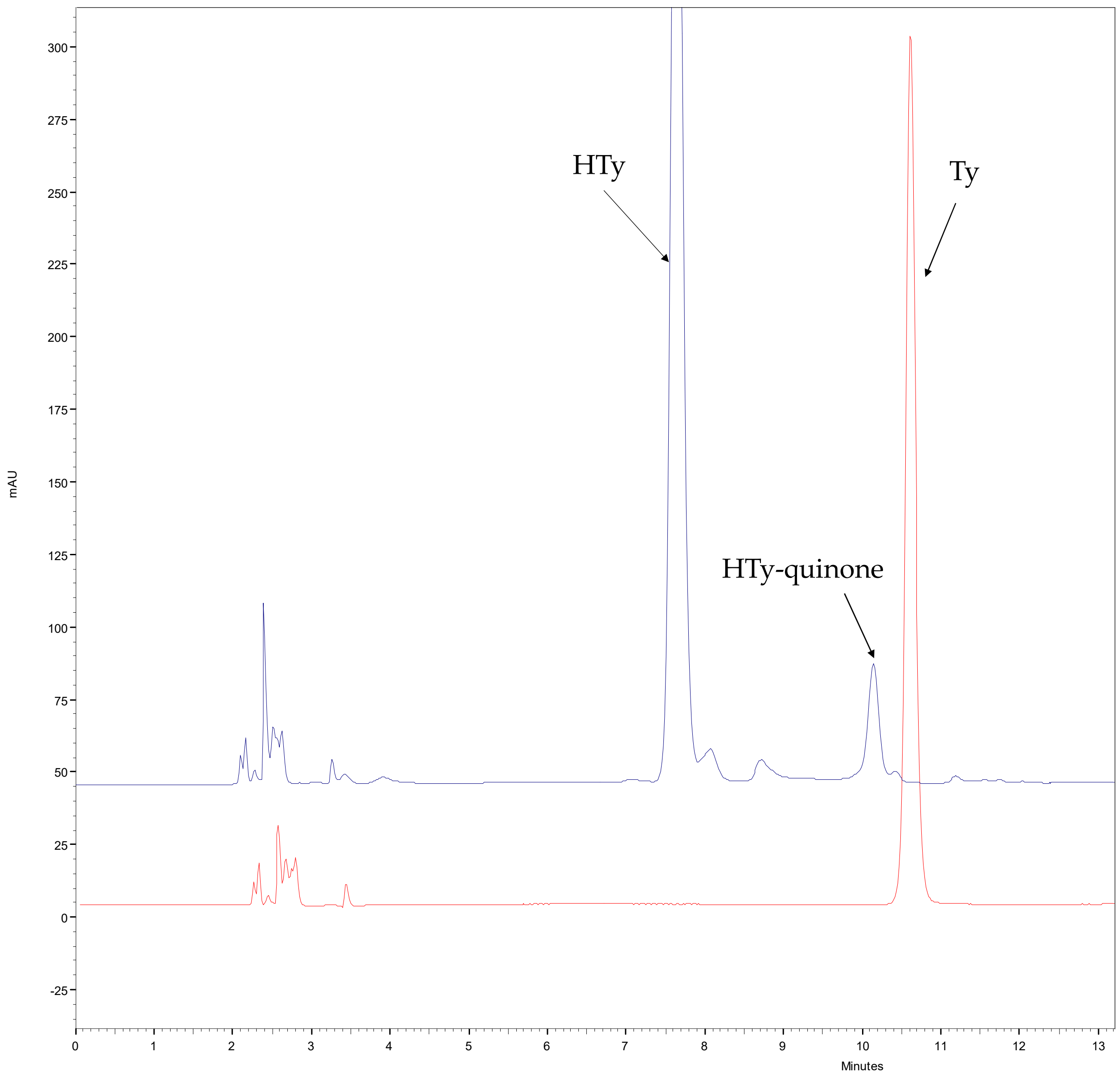

Figure S2. HPLC activity assays of OePPO2 with hydroxytyrosol (blue) and tyrosol (red).

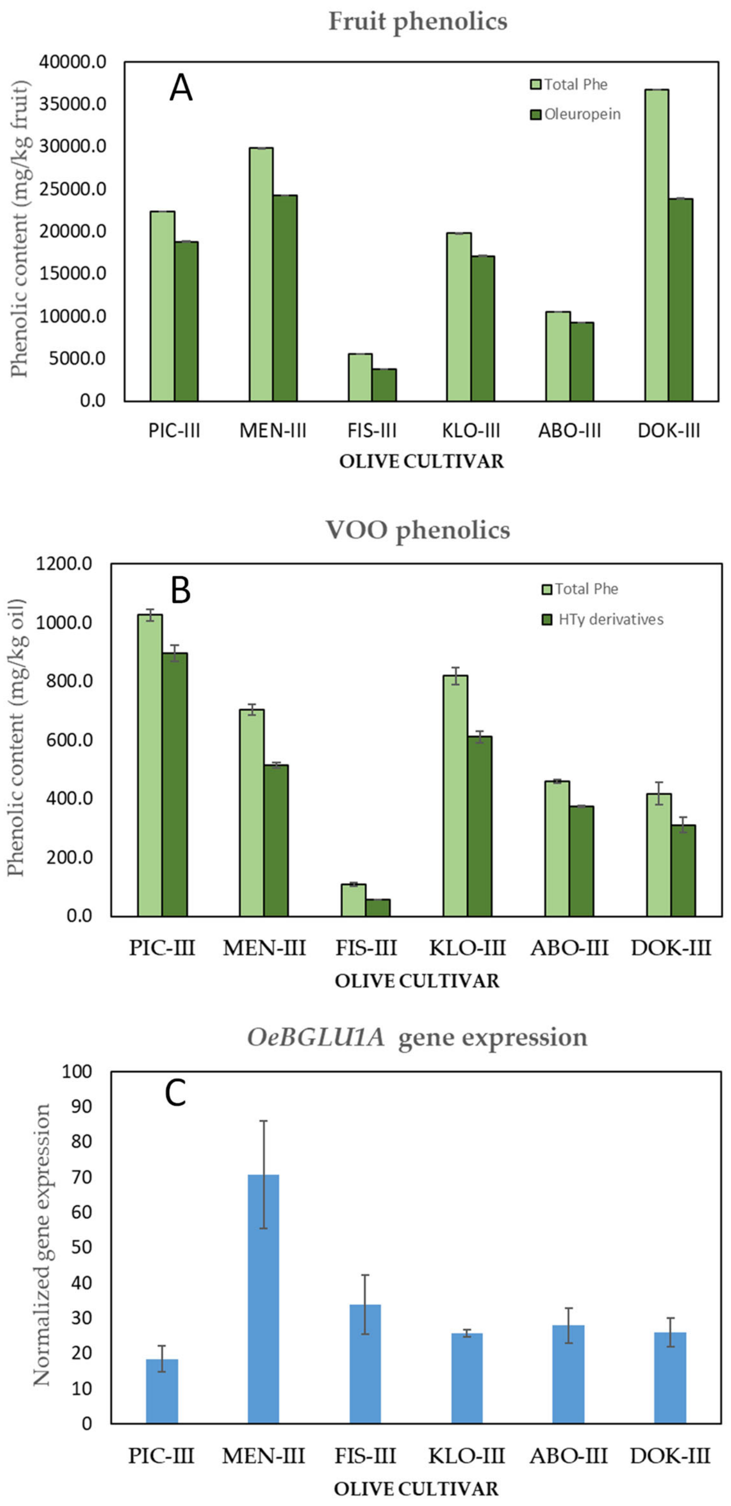

Figure S3. Correlation between the phenolic content of olive fruits (stage III) from 'Picual', 'Menya', 'Fishomi', 'Klon', 'Abou kanani', and 'Dokkar' cultivars (A), the phenolic content of their respective oils (B), and the relative expression levels of *OeBGLU1A* (C).

Table S1. Expression levels (FPKM) of the putative PPO transcripts (CNAG Olive Genome Data Base, OE6.OLIVEFAT) of seven olive cultivars. Ripening stage: II, yellow-green fruits (22–25 weeks-after flowering, WAF); IV, fully ripe fruits (35–40 WAF).

**Transcript**	**Mean**	**Fishomi (II/IV)**	**Picual (II/IV)**	**Menya (II/IV)**	**Piñonera (II/IV)**	**Klon (II/IV)**	**Dokkar (II/IV)**	**Abou Kanani (IV)**
OE6A114203	1163	15 / 13	13 / 19	551 / 2627	2904 / 1364	873 / 132	2961 / 3634	15
OE6A068152	818	877 / 394	871 /154	2728 / 507	400 / 289	3315 / 331	368 / 246	153
OE6A046766	485	7 / 7	72 / 170	7 / 13	105 / 133	1131 / 2965	145 / 1547	7
OE6A058214	405	6 / 5	6 / 6	233 / 1097	5 / 7	445 / 64	1505 / 1875	6
OE6A063859	159	44 / 142	43 / 110	63 / 161	37 / 54	352 / 839	9 / 14	196
OE6A110596	87	4 / 8	127 / 29	4 / 7	306 / 52	442 / 49	44 / 36	18
OE6A053284	70	67 / 21	74 / 41	259 / 48	120 / 25	184 / 31	17 / 5	14

Table S2. Identity percentage (%) between the main PPO transcripts (CNAG Olive Genome Data Base, OE6.OLIVEFAT). GenBank Accession Numbers in parenthesis. Gene given names in brackets.

**Transcript**	**Protein identity (%)**			
	OE6A068152 (MW038828) [OePPO1]	OE6A114203 (MW038829) [OePPO2]	OE6A046766 (OL870608) [OePPO3]	OE6A110596 (OM460173) [OePPO4]
OE6A068152				
OE6A114203	56.4			
OE6A058214	56.9	98.3		
OE6A046766	49.0	47.4		
OE6A063859	49.5	47.3	99.7	
OE6A110596	39.9	40.2	45.7	
OE6A053284	39.9	40.2	45.8	100

Table S3. Accession numbers of proteins used for phylogenetic analysis.

Identifier	GenBank	UniProtKB/Swiss-Prot.	NCBI Ref.	Organism
OePPO1	MW038828			*Olea europaea*
OePPO2	MW038829			*Olea europaea*
OePPO3	OL870608			*Olea europaea*
OePPO4	OM460173			*Olea europaea*
OE6A058214				*Olea europaea*
OE6A063859				*Olea europaea*
OE6A053284				*Olea europaea*
AmAS1	BAB20048			*Antirrhinum majus*
CgAUS1	AHN09736			*Coreopsis grandiflora*
CgAUS2a	AGT28745			*Coreopsis grandiflora*
CgAUS2b	AGT28746			*Coreopsis grandiflora*
IbCO	AAW78869			*Ipomoea batatas*
JrPPO1	ACN86310			*Juglans regia*
ToPPO1	ABX09994			*Taraxacum officinale*
ToPPO2	CAQ76694			*Taraxacum officinale*
ToPPO3	CBZ41490			*Taraxacum officinale*
ToPPO4	CBZ41491			*Taraxacum officinale*
ToPPO5	CBZ41492			*Taraxacum officinale*
ToPPO6	CCA94610			*Taraxacum officinale*
ToPPO7	CCD61123			*Taraxacum officinale*
ToPPO8	CCD61124			*Taraxacum officinale*
ToPPO9	CCD61125			*Taraxacum officinale*
ToPPO10	CCE45701			*Taraxacum officinale*
ToPPO11	CCD61126			*Taraxacum officinale*
VvPPOg	AAB41022			*Vitis vinifera*
LtLH	AAQ67412			*Larrea tridentata*
MdPPO1			NP_001306190	*Malus domestica*
MdPPO2	AAK56323			*Malus domestica*
MdPPO3	BAA21676			*Malus domestica*
TpPPO1	AAK13242			*Trifolium pratense*
TpPPO2	AAK13243			*Trifolium pratense*
TpPPO3	AAK13244			*Trifolium pratense*
TpPPO4	ABM68051			*Trifolium pratense*
TpPPO5	ABM68052			*Trifolium pratense*
TaPPO-A1a	ABK62801			*Triticum aestivum*
VfPPO-A1		Q06215		*Vicia faba*
StPPOA		Q41427		*Solanum tuberosum*
StPPOC		Q41428		*Solanum tuberosum*
SlPPOA		Q08303		*Solanum lycopersycum*
SlPPOE		Q08307		*Solanum lycopersycum*

Table S4. Molecular characteristics of the olive polyphenol oxidases.

Olive gene	Coding sequence (bp)	Protein (amino acids)	MW (kDa)	pI	Signal Peptide (amino acids)	Mature protein (amino acids)
OePPO1	1758	585	65.8	6.5	92	493
OePPO2	1764	587	66.1	6.3	93	494
OePPO3	1755	584	65.7	6.2	84	500
OePPO4	1755	584	66.1	6.0	98	486

Table S5. Oligonucleotides used for RT-qPCR.

Name	Sequence (5´-3´)	Amplicon size (bp)	Primer pair efficiency
qOePPO1-F	AGTGTACTGCTGCCGGAAGTTTG		
qOePPO1-R	TGTTGATTTGGAACCAACCACCT	146	101.4
qOePPO2-F	CGCCTGCTGTTCTTGTTTTCTCA		
qOePPO2-R	GTGCAAAGTGCAACCATCGATTA	169	91.8
qOePPO3-F	CGATGAAGAAAGTACGCCGGA		
qOePPO3-R	ACCAAAGTCACCAAAACATGCT	168	105.9
qOeBGLU1A-F	GAAGAACGTCGTAAAAGGCT		
qOeBGLU1A-R	CAACAATGACATGACTTTTCAACC	150	93.1
qOeEF1α-F	TGCTCTATCTGGATTGCCATT		
qOeEF1α-R	TCAAATGCCACCATGACTTC	107	94.8
qOeGAPDH-F	TGAGATGCTGCACAATGGTT		
qOeGAPDH-R	CACGATAGGCTTACGCAACA	131	95.2
